# Prominent coagulation disorder is closely related to inflammatory response and could be as a prognostic indicator for ICU patients with COVID-19

**DOI:** 10.1007/s11239-020-02174-9

**Published:** 2020-08-06

**Authors:** Yang Liu, Weibo Gao, Wei Guo, Yang Guo, Maojing Shi, Guiying Dong, Qinggang Ge, Jihong Zhu, Jin Lu

**Affiliations:** 1 Peking University People’s Hospital, Peking University Institute of Hematology, National Clinical Research Center for Hematologic Disease, No. 11 Xi-Zhi-Men South Street, Beijing, 100044 China; 2 Emergency Department, Peking University People’s Hospital, No. 11 Xi-Zhi-Men South Street, Beijing, 100044 China; 3 Trauma Center, Peking University People’s Hospital, Beijing, 100044 China; 4 Department of Critical Care Unit, Peking University Third Hospital, Beijing, 100191 China

**Keywords:** COVID-19, SARS-CoV-2, Blood coagulation, Inflammation, Anticoagulation

## Abstract

The new outbreak of Coronavirus Disease 2019 (COVID-19) has emerged as a serious global public health concern. A more in-depth study of blood coagulation abnormality is needed. We retrospectively analyzed 147 consecutive patients with COVID-19 who were admitted to three ICUs in Wuhan from February 9th, 2020 to March 20th, 2020. The baseline coagulation and other characteristics were studied. Our results showed that the prolonged PT, FDP, DD were positively correlated with the levels of neutrophils, ferritin, LDH, total bilirubin, multi-inflammation cytokines, and negatively correlated with the lymphocytes level (p < 0.01). The level of ATIII was significantly negatively correlated with the levels of neutrophils, ferritin, LDH, total bilirubin, IL2R, IL6 and IL8 (p < 0.05). The patients in the ARDS group had a more prominent abnormality in PT, FDP, DD and ATIII, while the patients in the AKI group had more prolonged PT, more severe FDP and DD level, more inferior ATIII and Fib level than those in the non-AKI group (p < 0.01). The value of PT, DD and FDP were positively correlated with the classical APACHE II, SOFA and qSOFA scores, while the ATIII was negatively correlated with them (p < 0.001). The high levels of PT, FDP and DD were correlated with in-hospital mortality (p < 0.001). In conclusion, blood coagulation disorder was prominent in ICU patients with COVID-19 and was correlated with multi-inflammation factors. The abnormality of blood coagulation parameters could be an adverse prognostic indicator for ICU patients with COVID-19.

**Electronic supplementary material:**

The online version of this article (10.1007/s11239-020-02174-9) contains supplementary material, which is available to authorized users.

## Highlights

- The blood coagulation disorders are prominent in ICU patients with COVID-19, which was mainly represented in the intrinsic coagulation pathway, an increase of fibrinolysis products, and a decrease of innate antithrombin.
- The level of prothrombin time, fibrin/fibrinogen degradation products and d-dimer were correlated with mortality in-hospital.
- The aberration of blood coagulation was significantly associated with multiple inflammation indicators (multi-cytokine, ferritin, and CRP), the lymphocytes, total bilirubin, and LDH.
- The interaction between coagulation and inflammation needs to be seriously considered, and appropriate treatment is needed to break this vicious circle.

## Introduction

The infection of the severe acute respiratory syndrome coronavirus 2 (SARS-CoV-2), which was firstly reported by China [1], is now seriously endangering the public healthcare systems worldwide. A pandemic of the Coronavirus Disease 2019 (COVID-19) has been declared by the World Health Organization. The number of infected patients in the world is rapidly increasing and has exceeded 3 million, while the number of related deaths has reached more than 217,769 as of 1st May 2020 [2]. The current data analysis shows that the virus invasion leads to a series of reactions and multiple organ system involvements [3, 4]. Several reports elucidated the abnormalities of blood coagulation in patients with COVID-19 [5–7]. However, an in-depth study of blood coagulation abnormality, influencing factors, the relationship between blood coagulation and inflammation reaction, and the impact on prognosis is still needed. This study set out with the aim of assessing the importance of the blood coagulation system abnormalities in patients with COVID-19 and discussing the characteristics, influencing factors, and its prognosis.

## Materials and methods

### Patients and study design

Consecutive patients with confirmed COVID-19 who were admitted to three temporarily organized Intensive Care Units (ICUs) of the Zhongfaxincheng campus of Tongji Hospital, affiliated to Huazhong University of Science and Technology in the city of Wuhan from February 9th, 2020 to March 20th, 2020, were retrospectively analyzed. The hospital is one of the hospitals that were designated to receive the patients with confirmed SARS-CoV-2 infection, such that the patients whose condition had worsened and needed vital signs monitoring were transferred to these ICUs from other departments or hospitals for further treatment. These ICUs were run by the medical staff from the Peking University-affiliated hospitals. The diagnosis of COVID-19 was made according to the World Health Organization interim guidance and confirmed by the RNA detection of the COVID-19 in the clinical laboratory. The confirmation was made either from throat swab or sputum samples according to the WHO guidance. The clinical outcomes were monitored up to March 23th, 2020. This study was approved by the Peking University People’s hospital Committee and is in accordance with the Declaration of Helsinki.

The detailed clinical information of each patient was obtained by the physicians using a standard questionnaire after admission to the ICU. The clinical information included demographic data, medical history, comorbidities, symptoms, signs, and laboratory findings. In addition, we also collected the Acute Physiology and Chronic Health Evaluation II (APACHE II), Sepsis-related Organ Failure Assessment (SOFA), and quick SOFA scores of each patient. The in-hospital death was recorded.

The samples for the coagulation tests and other indexes were collected on admission. The complete blood count was measured using the Sysmex XN-9000 automatic hematology analyzer (Sysmex, Japan). The coagulation parameters, including the prothrombin time (PT), international normalized ratio (INR), fibrinogen (Fib), activated partial thromboplastin time (APTT), fibrin/fibrinogen degradation products (FDP), DD (d-dimer) and antithrombin III (ATIII) were performed using the Stago STA-R automatic blood coagulation analyzer (Stago, France). The biochemical indexes, lactate dehydrogenase (LDH) and ferritin were measured using the Roche Cobas 8000 automatic biochemical analyzer (Roche, Switzerland). The cytokines interleukin-2 receptor (IL-2R), -6, -8, -10 and tumor necrosis factor-α (TNF-α) were detected using the Roche Cobas e602 electrochemical luminescence analyzer (Roche, Germany). The diagnosis of disseminated intravascular coagulation (DIC) was made according to the International Society on Thrombosis and Haemostasis (ISTH) diagnostic criteria [8]. Acute kidney injury (AKI) was diagnosed based on the KDIGO criteria [9], while ARDS was defined according to the Berlin definition [10]. Coagulopathy was recorded if a 3-s extension of the prothrombin time or a 5-s or 10-s extension of the activated partial thromboplastin time occurred. Liver dysfunction was defined as an increase in the alanine aminotransferase (ALT) level of at least two times higher than the upper limit of normal. The upper limit of the normal of ALT was defined as ˃ 41 U/L. The definition of leukopenia, lymphopenia, neutropenia and thrombocytopenia was less than 4 × 10^9^/L, 1.0 × 10^9^/L, 1.0 × 10^9^/L and 100 × 10^9^/L, respectively. The definition of anemia was hemoglobin of less than 110 g/L for female patients or 120 g/L for male ones.

### Statistical analysis

Continuous variables were expressed as medians and interquartile range (IQR), while categorical variables were summarized as counts and percentages. Variables were compared by the Mann–Whitney U test for continuous variables, while χ^2^ test or a Fisher’s exact test was used for categorical variables. All the calculations of the data correlation were performed with Spearman correlation analysis. Univariate linear regression analyses were conducted to identify the risk factors that are associated with the coagulation parameter. All factors with p values less than 0.10 were included in the multivariate linear regression. A two-sided p value of less than 0.05 was considered statistically significant. All the analyses were performed using the SPSS software version 23.0 (SPSS, Inc, Chicago, IL, USA).

## Results

### Baseline clinical characteristics of patients

Among all the 147 patients, the median age at baseline was 66 years (IQR 54–72), with a male percentage of 54.4% (Table 1). The median time from symptom onset to diagnosis was 12 days. The median APACHE II, SOFA and qSOFA scores were 11 (IQR 6–17), 2 (IQR 1–4) and 0 (IQR 0–1), respectively. A total of 35 (23.8%) of the patients died during their hospital stay. Table 1 showed the differences in clinical manifestations, comorbidities, physical signs, and laboratory tests between the survivors and the non-survivors. These differences included age, blood coagulation parameters, blood cell count, liver and hepatic dysfunction, vital physical signs, and critical illness score.

**Table 1:** Baseline clinical characteristics

Parameters	Total	Survivors N = 112	Non-survivors N = 35	p-value
Age (year)	66 (54–71)	64 (52–72)	69 (60–78)	0.016
Sex (male %)	54.4%	51.8%	62.9%	0.251
Time from symptom onset to diagnosis (days)	12 (8–16)	13 (8–16)	11 (8–14)	0.258
Coagulation disorder				
PT (s)	14.3 (13.7–15.4)	14.0 (13.5–14.6)	15.9 (14.8–18.2)	< 0.001
APTT (s)	41.0 (36.4–46.2)	40.6 (36.4–45.7)	42.4 (37–49.9)	0.256
Fib (g/L)	5.0 (3.7–6.2)	5.0 (4.1–6.1)	4.9 (2.9–6.8)	0.731
DD (mg/L)	1.8 (0.7–5.5)	1.3 (0.5–2.7)	7.8 (2.5–21.0)	< 0.001
FDP (g/L)	5.9 (4.0–22.8)	4.8 (4.0–10.0)	70.8 (9.6–150.0)	< 0.001
AT III (%)	94.0 (79.8–106.3)	96.0 (85.0–108.0)	80.0 (67.0–95.0)	0.001
ISTH DIC score ≥ 5	23 (15.6%)	2 (1.8%)	21 (57.1%)	< 0.001
Blood cell abnormality (median, range)				
WBC (*10^9^/L)	6.2 (4.5–10.1)	5.3 (4.3–7.1)	12.6 (7.3–17.2)	< 0.001
Lyc (*10^9^/L)	0.8 (0.6–1.3)	0.9 (0.7–1.4)	0.6 (0.4–0.7)	< 0.001
Neu (*10^9^/L)	4.5 (3.0–8.1)	3.8 (2.7–5.4)	11.4 (6.8–15.4)	< 0.001
Mono (*10^9/L)	0.5 (0.3–0.6)	0.5 (0.3–0.6)	0.4 (0.3–0.6)	0.671
Hb (g/L)	122 (113–137)	121 (113–135)	131 (110–145)	0.091
PLT(*10^9/L)	194 (139–277)	221 (148–294)	155 (111–215)	0.004
Total bilirubin (μmol/l)	10.1 (7.0–15.7)	8.9 (6.6–13.0)	17.6 (11.6–25.3)	< 0.001
Serum creatinine (μmol/l)	80 (60–103)	82 (61.5–156.3)	70 (58–87)	0.030
Co-morbidity (%)				
Diabetes	31 (21.1%)	24 (21.4%)	7 (20%)	0.856
Hypertension	67 (45.6%)	51 (45.5%)	16 (45.7%)	0.985
Coronary artery disease	26 (17.7%)	17 (15.2%)	9 (25.7%)	0.154
Chronic obstructive pulmonary disease	19 (12.9%)	14 (12.5%)	5 (14.3%)	0.783
Onset temperature (%)				
> 39.0	29 (19.7%)	22 (19.6%)	6 (17.1%)	0.742
38.1 to 39.0	62 (42.2%)	46 (41.4%)	18 (51.4%)	0.281
37.3 to 38.0	33 (22.4%)	26 (23.2%)	7 (20%)	0.691
< 37.3	23 (15.6%)	18 (16.1%)	4 (11.4%)	0.597
Onset symptoms (%)				
Cough	84.4%	84.8%	82.9%	0.780
Expectoration	44.9%	45.5%	42.9%	0.781
Chest tightness/shortness of breath	63.9%	62.5%	68.6%	0.514
Fatigue	44.2%	48.2%	31.4%	0.081
Diarrhea	27.2%	29.5%	20%	0.272
Nausea/vomiting	28.6%	31.3%	20%	0.198
Consciousness disorder	37.4%	18.8%	97.1%	< 0.001
Heart rate on admission	99 (86–109)	95 (86–105)	109 (97–120)	< 0.001
Respiratory rate on admission	26 (23–30)	25 (21–29)	30 (26–33)	< 0.001
Mean arterial pressure on admission	98 (89–109)	99 (90–109)	96 (77–108)	0.217
SpO2 on admission	92% (87%-96%)	94% (90%-96%)	86% (80%-88%)	< 0.001
APACHE-II	11 (6–17)	8 (6–10)	19 (18–22)	< 0.001
SOFA	2 (1–4)	1 (1–2)	5 (4–5)	< 0.001
qSOFA	0 (0–1)	1 (0–1)	1 (1–2)	< 0.001

*PT* prothrombin time, *APTT* activated partial thromboplastin time, *Fib* fibrinogen, *DD* dimer, *FDP* fibrin/fibrinogen degradation products, *AT* antithrombin, *WBC* white blood cell, *Lyc* lymphocyte, *Neu* neutrophil; *Mono* monocyte, *Hb* hemoglobulin, *PLT* platelet, *APACHE-II* Acute Physiology and Chronic Health Evaluation II, *SOFA* the Sepsis-related Organ Failure Assessment, *qSOFA* quick SOFA

At the time of diagnosis, the percentage of a prolonged prothrombin time by more than 3 s and 6 s was 15.6% and 4.1%, respectively. The percentage of a prolonged APTT by more than 5 s and 10 s was 36.7% and 16.3%, respectively. The elevated fibrinolysis related markers FDP (more than 20 g/L) and DD (more than 0.5 mg/L) account for 23.8% and 82.3%, respectively. The average level of intrinsic ATIII was 93.2% (range: 48–129%). At the time of diagnosis, the percentage of leukopenia, lymphopenia, neutropenia, thrombocytopenia and anemia was 16.3%, 1.4%, 59.2% 12.2% and 25.22%, respectively.

According to the International Society on Thrombosis and Haemostasis (ISTH) diagnostic criteria for disseminated intravascular coagulation (DIC), 23 cases among all the cohort (15.6%) matched the overt DIC (score ≥ 5). Twenty cases (57.1%) of the non-survivors matched the overt DIC criteria in the course of the disease. However, only two survivors fulfill the DIC criteria during the hospital stay, and the incidence is significantly lower than that in the non-survivor group (p < 0.001).

### Correlation between blood coagulation aberrations and other laboratory indexes, ARDS, AKI, hepatic abnormality

As shown in Supplementary Table 1, the prolonged PT, FDP and d-dimer were positively correlated with the level of neutrophils, ferritin, LDH, total bilirubin, and multi-inflammation cytokines, and negatively correlated with the lymphocytes level. The level of ATIII was significantly negatively correlated with the level of neutrophils, ferritin, LDH, total bilirubin, IL2R, IL6 and IL8. We did not find a correlation between APTT or Fib and other laboratory indicators.

As shown in Supplementary Table 2, the patients in the ARDS group had a more prominent abnormality in PT, FDP, DD and ATIII. The level of APTT and Fib did not show any differences between the ARDS and non-ARDS groups. The patients in the AKI group had more prolonged PT, more severe FDP and DD level and more inferior ATIII and Fib than those in the non-AKI group. The level of APTT was not significantly different between the AKI and non-AKI groups. On the contrary, the coagulation parameter, as mentioned above, did not differ between the hepatic abnormality and non-hepatic abnormality groups.

### Regression for blood coagulation parameter

As shown in Table 2, based on the model of multiple linear regression analysis, the IL6 (p = 0.020), IL8 (p = 0.008), LDH (p = 0.001) and neutrophils (p = 0.024) were independent risk factors that contributed to PT. IL2R (p = 0.009), TNF α (p = 0.008) and LDH (p < 0.001) were independent risk factors that contributed to d-dimer. Ferritin (p = 0.024), IL2R (p = 0.001) TNF α(p = 0.001), and LDH (p < 0.001) were independent risk factors that contributed to FDP.

**Table 2:** Results of multivariate linear regression analysis for PT

Coefficients	Unstandardized β	SE	Standardized β	t	p
IL2R	0.000	0.000	0.187	1.699	0.092
IL6	− 0.001	0.000	− 0.307	− 2.365	0.020
IL8	0.003	0.001	0.307	2.703	0.008
IL10	0.001	0.006	0.008	0.082	0.935
CRP	0.001	0.002	0.051	0.553	0.581
PCT	0.003	0.011	0.022	0.286	0.776
LDH	0.002	0.001	0.374	3.338	0.001
Ferritin	− 4.24E−5	0.000	− 0.081	− 0.856	0.394
Tbil	0.019	0.013	0.125	1.478	0.142
Neu	0.071	0.031	0.229	2.288	0.024
Lyc	− 0.385	0.254	− 0.115	− 1.515	0.133

*PT* prothrombin time, *IL* Interleukin, *CPR* C reactive protein, *PCT* procalcitonin, *LDH* lactate dehydrogenase, *Neu* neutrophil, *Lyc* lymphocyte

### Value of blood coagulation in predicting the in-hospital mortality

**Fig. 1:**
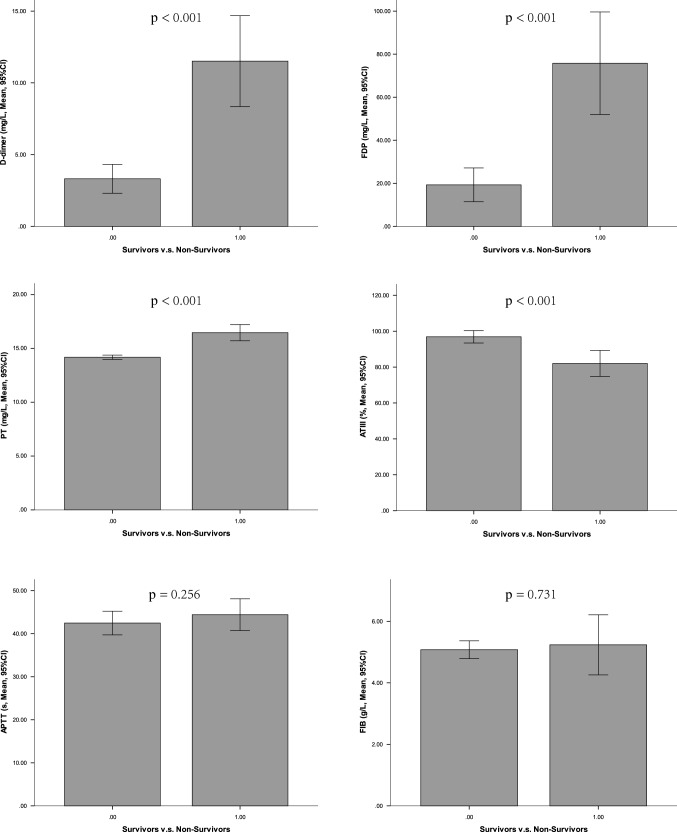
The blood coagulation dysfunction in survivors v.s. non-survivors. *PT* prothrombin time, *APTT* activated partial thromboplastin time, *Fib* fibrinogen, *DD* dimer, *FDP* fibrin/fibrinogen degradation products, *AT* antithrombin

**Fig. 2:**
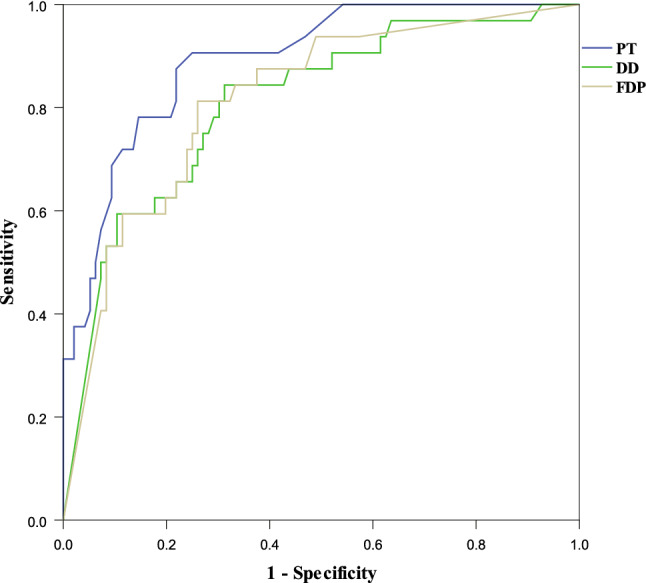
ROC curve for the coagulation parameter in predicting in-hospital mortality. *PT* prothrombin time, *DD* dimer, *FDP* fibrin/fibrinogen degradation products

The values of PT, DD and FDP were positively correlated with the classical APACHE II, SOFA, qSOFA scores, while ATIII was negatively correlated with them, which have shown to be important predictors for the in-hospital mortality in our cohort (p < 0.001). The Mann–Whitney U test and area under the Receiver Operating Characteristic (AUC) methods were used to assess the coagulation biomarker for the evaluation of mortality. As shown in Fig. 1 and Table 1, the results showed that the median PT value was 14.0 s (95% CI 12.6–16.5 s) in the survivors compared to 15.9 s (13.2–21.4 s) in the non-survivors (p < 0.001). Similarly, the median ATIII value was 96.0% (95% CI 70–125%) vs. 80.0% (95% CI 49–119%) (p < 0.001); the median d-dimer were 1.3 (95% CI 0.23–21.0) vs. 7.8 (95% CI 0.74–21.0) (p < 0.001) while the median FDP level was 4.8 (95% CI 4.0–150) vs. 70.8 (95% CI 4.0–150) (p < 0.001) in the survivor group and non-survivor group. Figure 2 shows that the AUC values for PT, FDP and DD were 0.892, 0.81 and 0.809, respectively. The optimal cut-off values for PT, FDP and DD were 14.6 s, 0.26 g/L and 2.0 mg/L, respectively. For both methods, we did not find an impact of ATT and Fib on the mortality.

## Discussion

In this retrospective cohort study, we found that the infection of SARS-CoV-2 can lead to prominent coagulation abnormalities. The blood coagulation dysfunction was mainly represented in the intrinsic coagulation pathway, an increase of fibrinolysis products, and a decrease of innate antithrombin. This dysfunction was significantly associated with multiple inflammation indicators (multi-cytokine, ferritin and CRP), lymphocytes, total bilirubin, and LDH. The mortality in non-survivors with coagulation disorder was superior to that in survivors.

Our results suggest that of all the routine coagulation tests, the PT, FDP and DD concentrations were prognostic biomarkers that are associated with inferior survival in our SARS-CoV-2 infected ICU population, while AT III was associated with superior survival, which provides useful tools in parallel with the APACHE II and SOFA scores for predicting the ICU mortality. Our results were consistent with recent reports for COVID-19 that blood coagulation abnormalities were common for severe patients and had adverse prognostic significance [4, 5]. Although the SOFA and APACHE II scores can predict mortality well, they are complex and time-consuming. Therefore, the current study provides useful coagulation information to evaluate the patients at high risk of death quickly.

Coagulation disorders could exist in patients with the viral infection. Our results were consistent with the recent literature about the COVID-19 [5]. The mechanisms of coagulation dysfunction in COVID-19 are complex. It may include the direct damage of endothelial cells, imbalance of inflammatory response, over-activation of the immune system, ischemia hypoxia reperfusion injury, drug factors, et al. The affinity of SARS-CoV-2 with Angiotensin-converting enzyme 2 (ACE 2) is 10–20 times than SARS [11, 12]. ACE2 was expressed in alveolar epithelial cells, arterial endothelial cells, small intestinal epithelial cells and immune tissues [13]. The tissue factor release is the initiation of the extrinsic coagulation pathway. Besides, the infection of SARS-CoV-2 overactivated the immune system, resulting in uncontrolled inflammatory damage [14]. Our results highlight the correlation between blood coagulation aberration and inflammation abnormality. The SARS-CoV-2 infection can cause an inflammation cascade, and the inflammatory reaction can initiate coagulation and reduce the natural anticoagulation mechanism and fibrinolysis system damage. Inflammatory cytokines are the main mediators and they participate in the activation of coagulation. Endogenous anticoagulants can inhibit the increase of cytokine level, weaken the response of cells to inflammatory mediators, promote the neutralization of some inflammatory mediators and reduce the loss of the endothelial barrier function. The downregulation of the anticoagulant pathway does promote not only thrombosis but also aggravates the inflammatory process. When the inflammation-coagulation interaction overwhelms the natural defense system, catastrophic events will occur and lead to a vicious circle. Coagulation disorders can also aggravate inflammation. For instance, the tissue factor-factor VIIa complex can induce pro-inflammatory effects on the macrophage/monocytes. The intrinsic antithrombin decreases both the tissue factor and IL-6 expression in the monocytes and endothelium [15].

We hypothesized that the hyperactive coagulation functions of the patients might be related to the systemic inflammation. Extensive cross-linking existed between the coagulation system and the immune and inflammatory system. For instance, our results showed that coagulation dysfunction was correlated with AKI, which was associated with a systemic inflammatory syndrome. In critically ill patients with AKI, the inflammatory cytokines are markedly elevated and associated with significant and clinically meaningful increases in the risk of death [16]. Similarly, neutrophils and lymphocytes were influenced by in vivo inflammation. The NLR (neutrophil to lymphocyte ratio) is a potential inflammation marker in many diseases [17].

The crosstalk between coagulation and inflammation can significantly affect disease progression and lead to a poor outcome. This interaction needs to be seriously considered, and appropriate treatment is needed to break the vicious circle. In addition to the mortality prediction value, the coagulation parameters may also have important therapeutic implications since the coagulation-inflammation interaction may play a role in the course of the disease. It is very complicated to control inflammation without effective antiviral drugs, thus inhibiting excessive blood coagulation reaction is practicable. On the other hand, one of the possible mechanisms of poor prognosis in patients with coagulation disorder is the presence of micro thromboses, such as pulmonary venous thromboembolism exacerbating ventilation-perfusion mismatch [18]. Thus, anticoagulation may play a key role in the treatment. Recently, the ISTH published an interim guideline on recognition and management of coagulopathy in COVID-19 and proposed a monitoring and treatment flow chart [19]. They recommended measuring d-dimers, PT, and platelet in all patients, and if there is a worsening of these parameters, more aggressive critical care support is warranted. Tang et al. suggested that patients meeting “sepsis-induced coagulopathy” criteria or with markedly elevated d-dimer may benefit from low-molecular-weight heparin anticoagulant therapy [20]. Similarly, Taisheng Li et al. proposed a low-molecular-weight heparin anticoagulant therapy for severe type SARS-CoV-2 infected patients to break out the imbalance of coagulation [21]. They proposed that the anticoagulation therapy is recommended for the COVID-19 patients when the d-Dimer value is four times higher than the normal upper limit, except for the patients with anticoagulant contraindications.

The lack of a more detailed evaluation of the coagulation parameters (e.g., Protein C, Protein S, plasminogen activator, plasminogen activator inhibitor, tissue factor pathway inhibitor, and Thrombomodulin) and vascular endothelium injury was an inherent limitation of this work. However, our study still enriches our understanding of the pandemic SARS-CoV-2 from the unique coagulation point of view.

In conclusion, blood coagulation disorders are prominent in ICU patients with COVID-19. Coagulation dysfunction was correlated with multi-inflammation factors. PT, d-dimer, FDP, and ATIII were found to be predictions of mortality. Breaking the coagulation-inflammation vicious interaction may be a useful aid for establishing an accurate therapeutic strategy and preventing disease progression, reducing mortality.

## Electronic supplementary material

Below is the link to the electronic supplementary material.

Supplementary file1 (DOCX 17 kb)

Supplementary file2 (DOCX 14 kb)

## Data Availability

Please contact the authors for data requests.

## Abbreviations

COVID-19: Coronavirus Disease 2019
SARS-CoV-2: Severe acute respiratory syndrome coronavirus 2
ICU: Intensive Care Unit
APACHE II: Acute Physiology and Chronic Health Evaluation II
SOFA: Sepsis-related Organ Failure Assessment
PT: Prothrombin time
INR: International normalized ratio
Fib: Fibrinogen
APTT: Activated partial thromboplastin time
FDP: Fibrin/fibrinogen degradation products
AT III: Antithrombin III
DD: d-Dimer
LDH: Lactate dehydrogenase
IL: Interleukin
TNF-α: Tumor necrosis factor
CRP: C-reactive protein
PCT: Procalcitonin
DIC: Disseminated intravascular coagulation
ISTH: International society on thrombosis and haemostasis
AKI: Acute kidney injury
ARDS: Acute respiratory distress syndrome
ALT: Alanine aminotransferase
IQR: Interquartile range
NLR: Neutrophil to lymphocyte ratio
