# Supplementary material for: Prominent coagulation disorder is closely related to inflammatory response and could be as a prognostic indicator for ICU patients with COVID-19
Source: J Thromb Thrombolysis. 2020 Aug 6;50(4):825–32. doi: 10.1007/s11239-020-02174-9 (PMC7408978; doi:10.1007/s11239-020-02174-9)
Supplement: Supplementary file 1 — Supplementary file1 (DOCX 17 kb) [file 11239_2020_2174_MOESM1_ESM.docx]

Suppl Table 1. The correlation between blood coagulation parameters and other laboratory indexes

|  | CRP | PCT | LDH | Ferritin | Neu | Lyc | IL2R | IL6 | IL8 | IL10 | TNF-α | C3 | C4 | Tbil | Scr | APACHE II | SOFA | qSOFA |
| --- | --- | --- | --- | --- | --- | --- | --- | --- | --- | --- | --- | --- | --- | --- | --- | --- | --- | --- |
| PT | .513 | .539 | .491 | .467 | 0.561 | -.412 | .449 | .410 | .294 | .307 | .239 | -.020 | -.211 | 0.477 | -0.044 | .474 | .544 | .340 |
| p | .000 | .000 | .000 | .000 | .000 | .000 | .000 | .000 | .001 | .000 | .005 | .845 | .035 | .000 | 0.598 | .000 | .000 | .000 |
| PT-INR | .500 | .514 | .448 | .426 | 0.537 | -.384 | .449 | .405 | .284 | .288 | .240 | -.031 | -.217 | 0.434 | -0.036 | .448 | .511 | .323 |
| p | .000 | .000 | .000 | .000 | .000 | .000 | .000 | .000 | .001 | .001 | .005 | .756 | .030 | .000 | 0.669 | .000 | .000 | .000 |
| FDP | .463 | .562 | .647 | .483 | 0.593 | -.426 | .443 | .462 | .425 | .255 | .393 | .066 | -.205 | 0.468 | -0.021 | .494 | .484 | .491 |
| p | .000 | .000 | .000 | .000 | .000 | .000 | .000 | .000 | .000 | .004 | .000 | .522 | .045 | .000 | 0.816 | .000 | .000 | .000 |
| DD | .497 | .573 | .667 | .488 | 0.601 | -.380 | .473 | .488 | .438 | .290 | .405 | .088 | -.201 | 0.418 | -0.094 | .505 | .501 | .511 |
| p | .000 | .000 | .000 | .000 | .000 | .000 | .000 | .000 | .000 | .001 | .000 | .382 | .045 | .000 | 0.260 | .000 | .000 | .000 |
| ATIII | -.311 | -.337 | -.251 | -.284 | -0.308 | .149 | -.286 | -.198 | -.265 | -.099 | -.140 | .144 | .199 | -0.281 | -0.018 | -.419 | -.287 | -.175 |
| p | .000 | .000 | .005 | .001 | .000 | .095 | .001 | .028 | .003 | .278 | .123 | .165 | .055 | 0.001 | 0.839 | .000 | .001 | .050 |
| APTT | .261 | .201 | .010 | .153 | -0.060 | -.084 | .092 | .145 | -.039 | .106 | .163 | .081 | .211 | -0.098 | 0.129 | .057 | .150 | .066 |
| p | .002 | .017 | .906 | .077 | 0.47 | .312 | .286 | .092 | .656 | .219 | .059 | .426 | .035 | 0.240 | 0.122 | .493 | .071 | .432 |
| Fib | .353 | .037 | .044 | .155 | 0.085 | -.099 | .062 | .249 | -.016 | .028 | .034 | .344 | .257 | -0.060 | 0.051 | -.019 | -.021 | .033 |
| p | .000 | .666 | .599 | .072 | 0.309 | .237 | .476 | .003 | .854 | .746 | .692 | .000 | .010 | 0.472 | 0.545 | .816 | .805 | .691 |

CPR, C reactive protein; PCT, Procalcitonin; LDH, lactate dehydrogenase; Neu, neutrophil; Lyc, lymphocyte; IL, Interleukin; TNF, tumor necrosis factor; C3, complement 3; C4, complement 4; Tbil, total bilirubin; Scr, serum creatinine; APACHE-II, Acute Physiology and Chronic Health Evaluation II; SOFA, the Sepsis-related Organ Failure Assessment; qSOFA, quick SOFA; PT, prothrombin time; APTT, activated partial thromboplastin time; Fib, fibrinogen; DD, dimer; FDP, fibrin/fibrinogen degradation products; AT, antithrombin;
