# Supplementary material for: Prominent coagulation disorder is closely related to inflammatory response and could be as a prognostic indicator for ICU patients with COVID-19
Source: J Thromb Thrombolysis. 2020 Aug 6;50(4):825–32. doi: 10.1007/s11239-020-02174-9 (PMC7408978; doi:10.1007/s11239-020-02174-9)
Supplement: Supplementary file 2 — Supplementary file2 (DOCX 14 kb) [file 11239_2020_2174_MOESM2_ESM.docx]

Suppl Table 2. The comparison of blood coagulation dysfunction in ARDS, AKI and Hepatic Abnormality group

|  | ARDS | | AKI | | Hepatic Abnormality | |
| --- | --- | --- | --- | --- | --- | --- |
|  | (-) | (+) | (-) | (+) | (-) | (+) |
| PT | 14.00  (13.50-14.50) | 15.90  (14.80-18.10) | 14.10  (13.60-15.10) | 16.70  (15.20-19.00) | 14.30  (13.65 – 15.55) | 15.60  (13.85 - 16.85) |
| p | 0.000 | | 0.000 | | 0.122 | |
| PT-INR | 1.07  (1.01-1.11) | 1.27  (1.16-1.49) | 1.08  (1.03-1.16) | 1.34  (1.20-1.57) | 1.09  (1.04 – 1.23) | 1.13  (1.04 - 1.29) |
| p | 0.000 | | 0.000 | | 0.379 | |
| FDP | 4.55  (4.00-7.88) | 70.75  (9.85-150.00) | 5.20  (4.00 – 13.80) | 85.60  (9.40 - 150.00) | 5.80  (4.00 – 18.00) | 11.00  (4.00 – 150.00) |
| p | 0.000 | | 0.002 | | 0.142 | |
| DD | 1.39  (0.54-2.57) | 18.58 (2.59-21.00) | 1.57  (0.59 – 3.28) | 21.00  (2.45 - 21.00) | 1.79  (0.68 – 5.51) | 2.77  (0.51 – 21.00) |
| p | 0.000 | | 0.000 | | 0.144 | |
| ATIII | 97.50  (85.25-109.5) | 79.50  (69.25-94.00) | 94.00  (82.00 – 108.00) | 78.00  (61.00 –100.00) | 94.00  (79.50 – 106.50) | 100.00  (73.50 – 106.00) |
| p | 0.000 | | 0.007 | | 0.913 | |
| APTT | 40.55  (35.85-44.50) | 41.85  (36.23-50.18) | 40.30  (35.80 – 44.70) | 43.00  (37.90 - 57.30) | 40.80  (36.15 - 45.85) | 40.60  (32.40 – 49.05) |
| p | 0.518 | | 0.166 | | 0.997 | |
| Fib | 5.07  (4.02-6.12) | 4.92  (2.74-6.87) | 5.19  (4.15- 6.20) | 2.98  (2.56 - 5.99) | 5.19  (3.79 – 6.20) | 2.98  (2.19 – 4.69) |
| p | 0.665 | | 0.049 | | 0.054 | |

PT, prothrombin time; APTT, activated partial thromboplastin time; Fib, fibrinogen; DD, dimer; FDP, fibrin/fibrinogen degradation products; AT, antithrombin; ARDS, acute respiratory distress syndrome; AKI, acute kidney injury.
